# Comprehensive analysis of ATF3 as a diagnostic and prognostic biomarker from pan-cancer to clear cell renal cell carcinoma

**DOI:** 10.1007/s12672-026-05113-x

**Published:** 2026-04-30

**Authors:** Hao Ling, Guangmei Wu, Yanzhu Hu

**Affiliations:** 1 Department of Surgery, Klinikum rechts der Isar, TUM School of Medicine and Health, Technical University of Munich, 81675 Munich, Germany; 2 Hainan Haiyi Medical Legal Identification Center, Hainan Medical University, Haikou, 571199 Hainan China

**Keywords:** ATF3, Biomarker, Kidney renal clear cell carcinoma, Prognosis, Single cell RNA sequencing

## Abstract

**Background:**

ATF3 was found to play a complex role in various cancers; however, its systematic function in kidney renal clear cell carcinoma (KIRC) and across pan-cancer contexts remained incompletely understood.

**Methods:**

A comprehensive evaluation of ATF3 expression, diagnostic efficacy, prognostic relevance, and its association with the tumor immune microenvironment was conducted across multiple cancer types using publicly available databases. In KIRC, ATF3 expression in cell lines was validated through real-time quantitative PCR, Western blot, and immunofluorescence tests. Assessments of immune cell infiltration and functional enrichment studies were also carried out. Ultimately, single-cell RNA sequencing (scRNA-seq) were implemented to clarify the role of ATF3 at the cellular level in KIRC.

**Results:**

ATF3 expression was observed to be downregulated in most cancers and was shown to possess diagnostic and prognostic value. Experimentally, ATF3 was confirmed to be downregulated in KIRC cell lines. In KIRC, higher ATF3 expression was connected with an improved outcome. Functional analyses indicated that ATF3 was involved in the IL-17 signaling pathway. The analysis demonstrated that, among seven immune cells with markedly varying infiltration levels, naive B cells and resting memory CD4 T cells were more prevalent in the ATF3 high expression cohort. ScRNA-seq analyses identified endothelial-afferent/efferent arterioles/descending vasa recta (AEAs/DVR) as the key cell, with ATF3 expression primarily detected during the early stage of AEAs/DVR differentiation.

**Conclusion:**

ATF3 was found downregulated in many cancers and proposed as a pan-cancer biomarker; in KIRC, its low level predicted poor outcome, indicating a potential immunotherapy target.

**Supplementary Information:**

The online version contains supplementary material available at 10.1007/s12672-026-05113-x.

## Introduction

Kidney renal clear cell carcinoma (KIRC) is the predominant subtype of renal cell carcinoma, representing approximately 70–80% of all instances [1]. In 2022, approximately 430,000 new cases were reported globally, with both morbidity and mortality continuing to rise [2]. Despite continuous advances in treatments including surgery, targeted therapy, and immune checkpoint inhibitors, the overall prognosis for patients with advanced KIRC remains poor, with existing regimens showing limited efficacy in some patients and frequent significant adverse reactions [3–5]. Moreover, the heterogeneity of KIRC leads to significant variations in patient responses to similar treatments, posing a formidable challenge to precision medicine [6]. Emerging technologies such as machine learning, multi-omics integration, and cross-disease genetic crosstalk analysis have provided crucial support for solving the challenges in precise diagnosis and treatment of KIRC, as well as for discovering new biomarkers and therapeutic targets [7, 8]. Therefore, by integrating bioinformatics analysis to elucidate the pathogenesis of KIRC and developing novel biomarkers are crucial for enhancing the accuracy of early diagnosis, optimizing prognostic stratification, guiding personalized treatment, and ultimately improving patient clinical outcomes.

Transcription Factor 3 (ATF3), a member of the ATF/CREB family, is activated in response to diverse cellular stresses—including oxidative stress, hypoxia, and inflammation—and participates in regulating multiple biological processes such as apoptosis, proliferation, and immune responses [9–13]. KIRC is essentially a metabolic disease characterized by a reprogramming of energetic metabolism [14–18]. In particular the metabolic flux through glycolysis is partitioned [19–22], and mitochondrial bioenergetics and OxPhox are impaired, as well as lipid metabolism [20, 23]. In addition, metabolic reprogramming plays an important role in chemoresistance and in determining the sensitivity of neoplastic cells to drugs and regulates many biological characteristics of renal cancer stem cells [24–26]. In this scenario ATF3 has an important role in regulating cancer metabolism. In the realm of oncology, ATF3 demonstrates a dual function, serving as both a tumor suppressor (such as hepatocellular carcinoma, renal carcinoma, lung cancer) and an oncogene (such as breast cancer, colorectal cancer, prostate cancer) [27, 28]. Gao et al. demonstrated that ATF3 inhibits the growth and metastasis of KIRC by suppressing the EGFR/AKT/GSK3β/β-catenin signaling pathway [29]. Consequently, a comprehensive investigation into the multifaceted roles of ATF3, particularly functional networks and immunomodulatory effects within KIRC, is warranted to bridge the existing knowledge gap and evaluate its translational potential.

Therefore, this study aims to systematically elucidate the function of ATF3 in KIRC and its pan-cancer significance. To this end, we adopted an integrated approach: first, analyzing ATF3 expression and prognostic significance across multiple cancers using public transcriptomic and clinical databases; second, experimentally validating its expression in KIRC cell lines via real-time quantitative polymerase chain reaction (RT-qPCR), western blot, and immunofluorescence; and third, employing functional enrichment, immune infiltration, and single-cell RNA-seq analyses to decipher its regulatory mechanisms within the KIRC microenvironment. This research is expected to establish ATF3 as a novel biomarker and therapeutic target for KIRC, providing a theoretical basis for precision diagnosis and treatment.

## Materials and methods

### Data collection

The survival and phenotypic data for The Cancer Genome Atlas-KIRC (TCGA-KIRC) cohort, which encompassed 533 KIRC and 81 control samples, were gathered via UCSC Xena (https://xenabrowser.net/datapages/). The single-cell RNA sequencing (scRNA-seq) cohort GSE159115 (GPL16791), which included renal tissue samples from 7 KIRC and 6 controls, was gathered via the Gene Expression Omnibus (GEO) (http://www.ncbi.nlm.nih.gov/geo/).

### Expression of ATF3 in pan-cancer

The TCGA and GTEx databases (the control cohort) were implemented to gauge the expression levels of ATF3 in different cancer types, and the disparities between tumor and control tissues were showed using box plot.

### Prognostic analysis

The diagnostic efficacy of ATF3 in various cancers was analyzed via receiver operating characteristic (ROC) curves. The median expression of ATF3 was implemented to sort patients into cohorts with high and low expression. Kaplan-Meier (KM) curves were plotted via the survival (v 3.4-0) [30] and survminer (v 0.4.9) [31] packages to evaluate the associations of ATF3 expression with overall survival (OS), progression-free interval (PFI), disease-free interval (DFI), and disease-specific survival (DSS). To look into the connections between ATF3 and patient prognosis, Univariate Cox analysis was implemented via the survival package.

### Genomic alterations and mutation burden analysis

The cBioPortal (http://www.cbioportal.org/) was implemented to examine the mutation characteristics of ATF3 in pan-cancer. Analysis was done on the connections between ATF3 expression and microsatellite instability (MSI) and tumor mutation burden (TMB) and visualized using radar plots.

### DNA mismatch repair and stemness analysis

Through published literature, five mismatch repair genes [32] and four DNA methyltransferases [33] were obtained, and correlations between these genes and AFT3 in pan-cancer were investigated. The associations between ATF3 expression and differentially methylated probes-based stemness index (DMPsi) [34] were evaluated.

### Tumor immune microenvironment analysis

The estimate algorithm (v 1.0.13) [35] was applied to calculate the correlations between ATF3 expression and ImmuneScore, StromalScore, and EstimateScore in 33 cancers. The expression associations between ATF3 and immune checkpoint genes (ICGs), chemokines and their receptors, immune stimulators, and immune inhibitors were also systematically explored.

### Cell culture

Human renal tubular epithelial cells (HK-2), KIRC cells (769-P and 786-O) were purchased via Immocell (Xiamen, China). Cells were grown in media (HK-2: DMEM/F12; 769-P and 786-O: 1640) supplemented with 10% FBS and 1% P/S. At 37 °C, cells were cultured in a 5% CO2 atmosphere.

### RT-qPCR

FastPure Complex Tissue/Cell Total RNA Isolation Kit (Vazyme, China) was implemented for total RNA isolation. Reverse transcription was implemented via ABScript III RT Master Mix for RT-qPCR with gDNA Remover (ABclonal, China). RT-qPCR was adopted via Genious 2X SYBR Green Fast RT-qPCR Mix (ABclonal, China). Expression levels were gauged via the 2-ΔΔCt technique, with GAPDH assisting as the internal reference. Table S1 displayed the primer sequences that were necessary.

### Western blot

The BCA kit (P0010; Beyotime) was implemented to quantify the total proteins that were isolated from the cells using RIPA lysis buffer. Following SDS-PAGE separation, protein samples were transferred to PVDF membranes. After 30 min of blocking with 5% non-fat milk, the membranes were incubated for the whole night at 4 °C with primary antibodies against ATF3 (1:500, ABclonal) and GAPDH (1:10,000, ABclonal). The membranes were incubated for 30 min at ambient temperature the next day via Goat anti-Rabbit IgG (H + L) Secondary Antibody, HRP (Invitrogen, 1:5,000). The proteins were showed via the ECL chemiluminescence system and quantified using ImageJ software.

### Immunofluorescence staining

After being sown in 6-well plates, the cells were cultivated for the whole night. Following a PBS wash, the cells were permeabilized for 10 min using 0.1% Triton X-100 and fixed for 30 min by 4% paraformaldehyde. The cells were blocked with 1% BSA for 30 min at room temperature before receiving primary antibody treatment for the whole night at 4 °C. Following a PBS wash, the cells were treated for one hour at room temperature with secondary antibodies before being stained for 5 min with DAPI. The same antibodies were used as those for Western blot. A fluorescent microscope was implemented to see the cells after they had been attached via an anti-fade reagent.

### Functional and immune infiltration evaluation of ATF3 in KIRC

In the TCGA-KIRC cohort, based on the ideal cutoff value of ATF3 expression, patients were split into cohorts with high and low expression. KM curves were plotted to gauge prognostic disparities between cohorts. The associations between ATF3 expression and clinicopathological traits (age, gender, stage, and T/M/N stage) were further analyzed. The DESeq2 program (v 1.38.3) [36] was implemented to recognize differentially expressed genes (DEGs) among ATF3 expression cohorts, with |log_2_fold change (FC)| > 0.5 and *P* < 0.05. Volcano and heatmap graphs were drawn via the ggplot2 (v 3.5.1) [37] and pheatmap (v 1.0.12) [38], respectively. Subsequently, analyses of Gene Ontology (GO) and Kyoto Encyclopedia of Genes and Genomes (KEGG) enrichment was carried out via the clusterProfiler (*P* < 0.05) (v 4.6.2) [39]. Using the “c2.cp.kegg.symbols.gmt” from the Gene Set Enrichment Analysis (GSEA) (http://www.gsea-MSigdb.org/gsea/msigdb), GSEA was performed, using log_2_FC values for ranking, with screening conditions of |NES| > 1 and adj.*P* < 0.05. The CIBERSORT algorithm (v 0.1.0) [40] was implemented to gauge immune infiltration, and disparities between groups were compared via the Wilcoxon test.

### Drug sensitivity analysis, drug prediction and molecular docking

The oncoPredict package (v 1.2) [41] was implemented to gauge half-maximum inhibitory concentration (IC_50_) values of drugs based on the Cancer Drug Sensitivity Genomics (GDSC) (https://www.cancerrxgene.org/). To figure out the variations in drug sensitivity between the cohorts with high and low expression, Wilcoxon tests were adopted. Potential drugs targeting ATF3 were predicted using the DGIdb database (http://dgidb.genome.wustl.edu/), and approved drugs were selected for molecular docking. The AlphaFold database (https://alphafold.com/) supplied the ATF3 protein structure, while the PubChem (https://pubchem.ncbi.nlm.nih.gov/) furnished the medicines’ 3D structures. Molecular docking was completed via PyMol and AutoDock Vina software.

### ScRNA-seq analysis

The Seurat tool (v 5.1.0) [42] was used to perform quality control on GSE159115, removing genes found in less than 200 cells and removing cells with nFeature_RNA ≥ 7,500, nCount_RNA ≥ 50,000, and mitochondrial gene proportion ≥ 20%. After normalization, the vst technique was implemented to choose the top 2000 highly variable genes. Principal component analysis was performed, and the first 30 principal components were chosen. FindNeighbors and FindClusters functions were implemented to cluster cells at a 0.1 resolution. For dimensionality reduction and visualization, we turned to Uniform Manifold Approximation and Projection (UMAP). Genes used as markers in the literature were used to classify the cells [43]. Potential doublets were recognized via the DoubletFinder (v 2.0.4) [44]. Cells with significant disparities in ATF3 expression between KIRC and controls, and those with trends consistent with ATF3 differences in KIRC cells, were defined as key cells. To deeply analyze the functions of cells and the dynamic changes of genes, the CellChat program (v 1.6.1) [45] was used to analyze the cell-cell communication networks, and the Monocle package (v 2.26.0) [46] was employed for pseudo-time trajectory analysis. To identify the genes that dynamically change along the pseudo-time trajectory and the driver genes that co-express with ATF3, the Spearman correlation coefficients between each gene expression and pseudo-time, as well as ATF3 expression were calculated. The screening criterion was set as the absolute value of the correlation coefficient > 0.7. In terms of visualization, a gene expression matrix was constructed and cells were sorted by pseudo-time. The expression values of each gene were smoothed (using smooth.spline, df = 3) and standardized by Z-score before being plotted as a heatmap using the ComplexHeatmap package; at the same time, a scatter plot of the top 20 co-expressed genes and Locally Estimated Scatterplot Smoothing (LOESS) curves were drawn using ggplot2 to visually present the changing trend of key genes along the pseudo-time.

### Statistical analysis

Bioinformatics analyses were conducted using R packages, with Wilcoxon tests employed to assess differences between groups. Data values obtained through experimental analyses were provided as mean ± standard deviation (SD). One-way ANOVA or unpaired two-tailed Student’s t-tests were implemented for statistical analysis. GraphPad software was implemented to analyze the data. *P* < 0.05 were deemed statistically significant.

## Result

### ATF3 was universally downregulated in pan-cancer and had diagnostic value

**Fig. 1:**
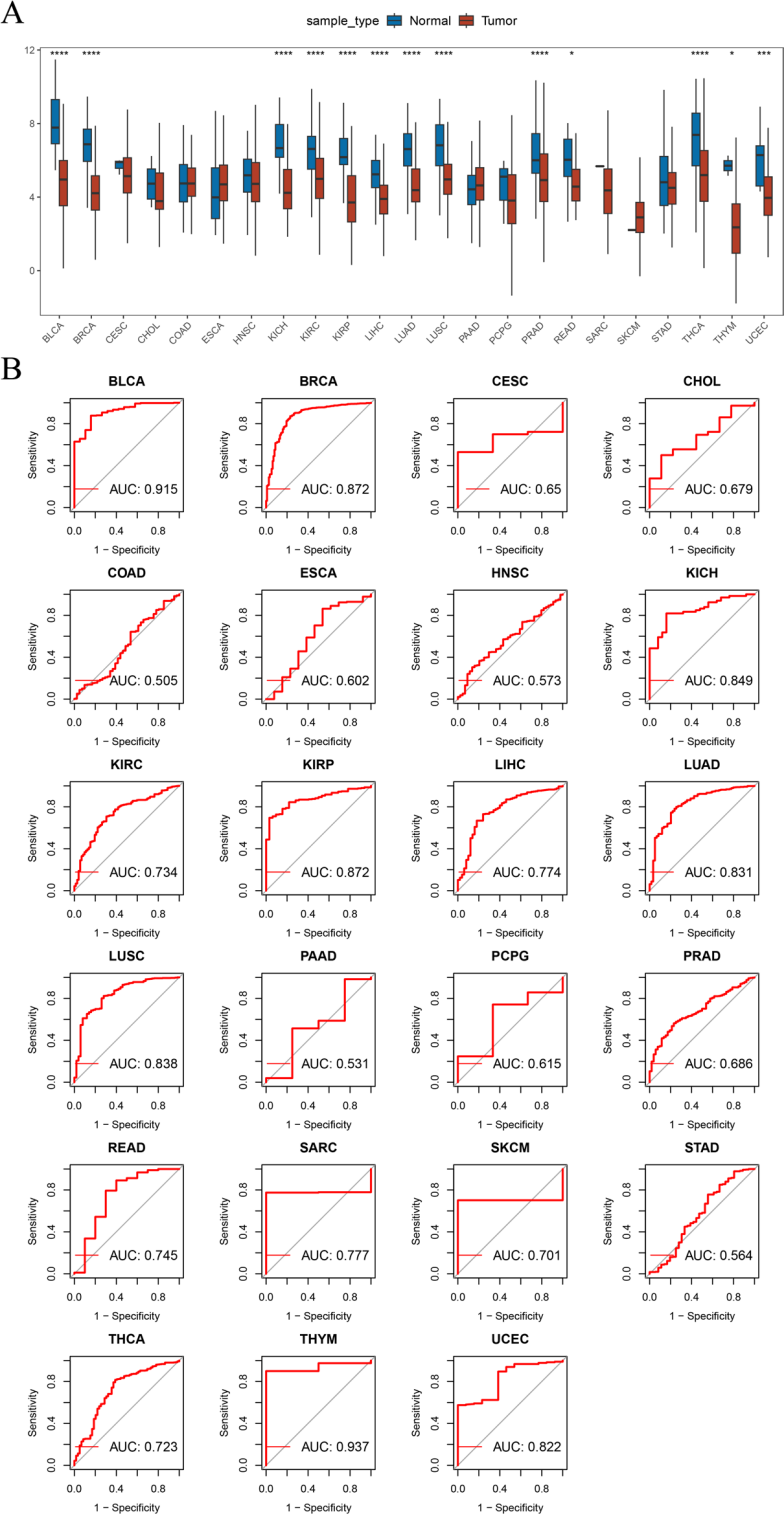
Expression and diagnostic significance of ATF3 in pan-cancer. **A** ATF3 expression in normal and cancer samples analyzed with the GTEx and TCGA datasets. **B** Receiver operating characteristic (ROC) analysis evaluated the diagnostic performance of ATF3 across cancer types. *, *P* < 0.05; ***, *P* < 0.001; ****, *P* < 0.0001

Pan-cancer analysis revealed that ATF3 was significantly downregulated in 13 cancer types (*P* < 0.05), including liver hepatocellular carcinoma (LIHC), kidney renal papillary cell carcinoma (KIRP), and KIRC (Fig. 1A). ROC analysis further assessed the diagnostic potential of ATF3 for different tumor types, showing good discrimination ability in 14 cancers (area under the curve (AUC) values > 0.7). Notably, ATF3 exhibited particularly high diagnostic efficacy in thymoma (THYM, AUC = 0.937) and bladder cancer (BLCA, AUC = 0.915), suggesting potential clinical application value in these tumor types (Fig. 1B). These findings indicated that the abnormal downregulation of ATF3 might be a common feature of various malignant tumors.

### ATF3 expression correlated with prognosis across multiple cancers

**Fig. 2:**
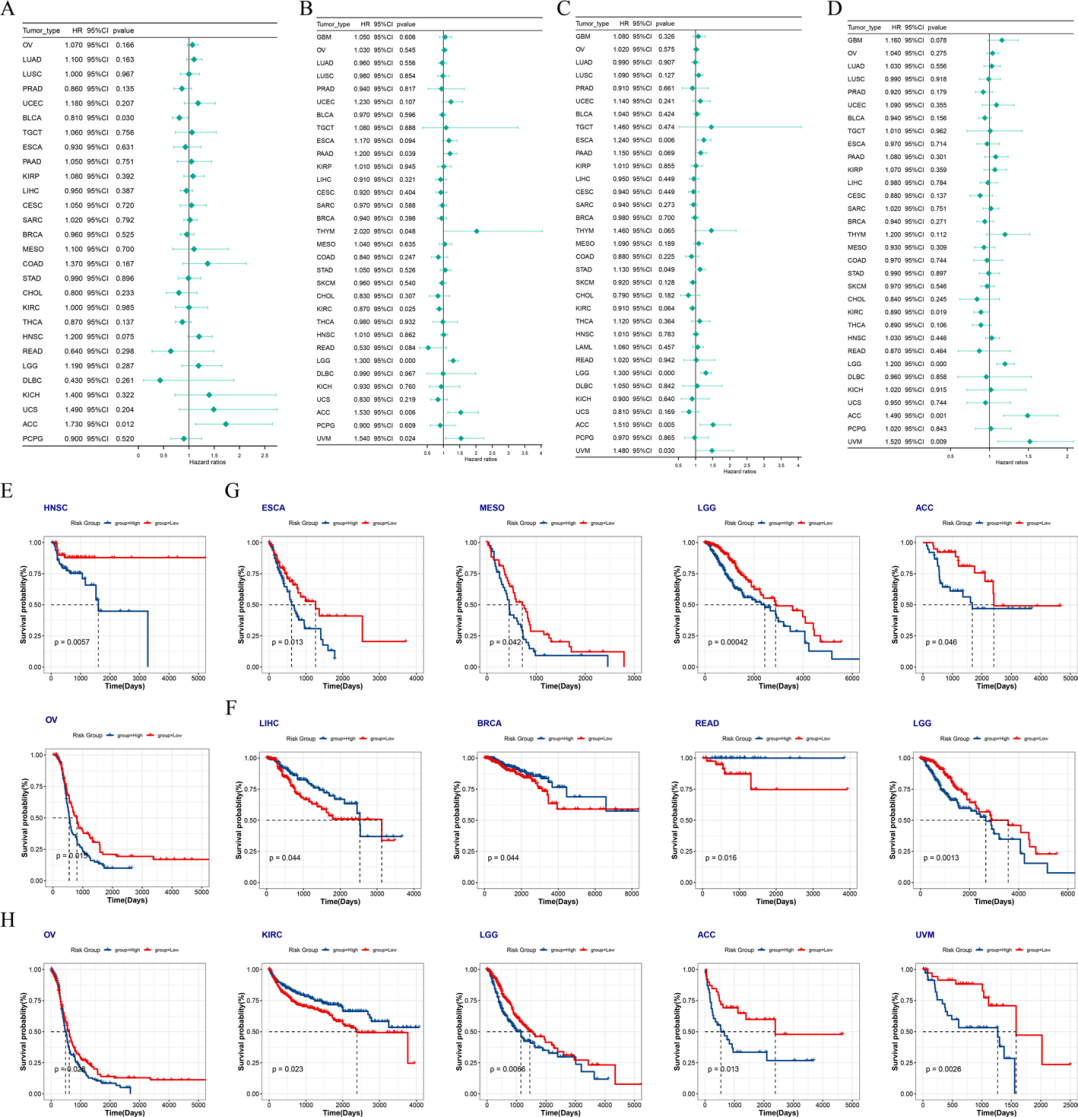
Prognostic evaluation of ATF3 in pan-cancer. **A**–**D** Univariate Cox regression analyses of ATF3 in different tumors for disease-free interval (DFI) (**A**), disease-specific survival (DSS) (**B**), overall survival (OS) (**C**) and progression-free interval (PFI) (**D**). **E**–**H** Patients were stratified by the median ATF3 expression; Kaplan-Meier curves compared the impact of high vs. low ATF3 expression on DFI (**E**), DSS (**F**), OS (**G**) and PFI (**H**) in each cancer type. HR, hazard ratio; CI, confidence interval

To judge the prognostic value of ATF3 in malignancies, ATF3 expression levels and survival outcomes was investigated. Univariate Cox analysis unveiled that in adrenocortical carcinoma (ACC), elevated ATF3 expression correlated with worse OS, DSS, DFI, and PFI. In low-grade glioma (LGG) and uveal melanoma (UVM), elevated ATF3 expression was substantially connected with worse OS, DSS, and PFI (Fig. 2A-D). KM analysis further corroborated these conclusions and revealed that elevated ATF3 expression was connected with poor DFI in head and neck squamous cell carcinoma (HNSC), poor DSS in LGG, poor OS in LGG, ACC, and PFI in LGG, ACC, and UVM (Fig. 2E-H).

### Mutation features of ATF3 and its complex associations with TMB/MSI

**Fig. 3:**
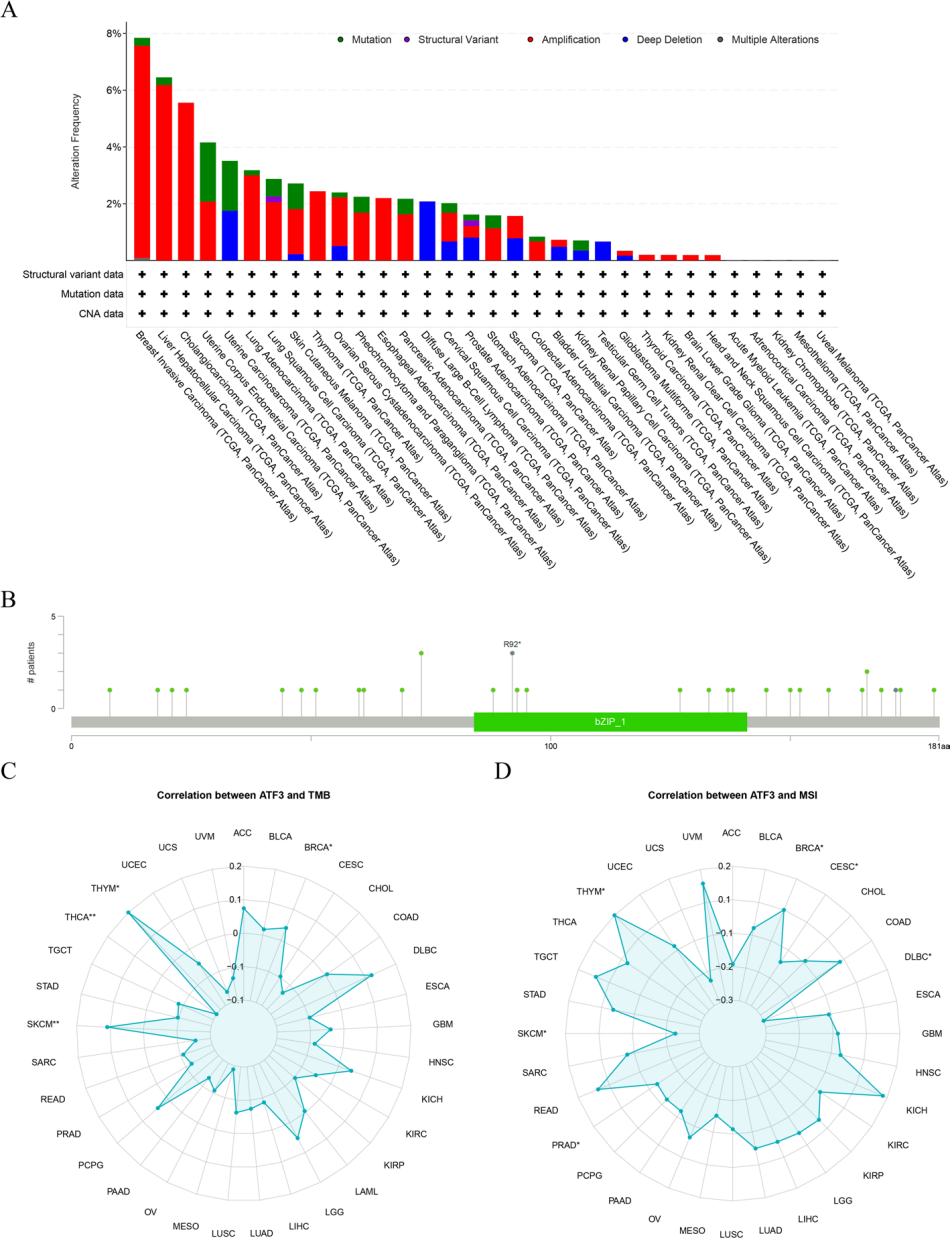
Mutation landscape and mutation-load analysis of ATF3 in pan-cancer. **A** Frequencies of distinct ATF3 mutation types across cancers. **B** Analysis showing that missense mutations (green) were the predominant ATF3 alteration. **C** Radar plot depicting the correlation between ATF3 expression and tumor mutation burden (TMB). **D** Radar plot depicting the correlation between ATF3 expression and microsatellite instability (MSI). *, *P* < 0.05; **, *P* < 0.01

Given that gene mutations significantly influence the regulation of cancer carcinogenesis and development, further exploration of ATF3’s genetic changes was conducted based on cBioPortal. ATF3 exhibited a higher frequency of genetic alterations in breast invasive carcinoma (BRCA), LIHC, and cholangiocarcinoma, primarily in the form of “amplification” (Fig. 3A). Mutation profiling identified that the dominant variant type was a missense mutation, and structural alteration of the R92* domain was likely to impair ATF3 function (Fig. 3B). Considering that TMB and MSI are significant predicted markers for immunotherapy efficacy, the correlations between ATF3 and these markers were further investigated. The findings pointed out a positive connection between TMB and MSI in THYM and BRCA and ATF3 expression (Fig. 3C-D). These findings pointed to a close ATF3-mutation relationship bearing on immunotherapy outcomes.

### ATF3 expression was closely connected to the tumor microenvironment (TME) and stemness characteristics

**Fig. 4:**
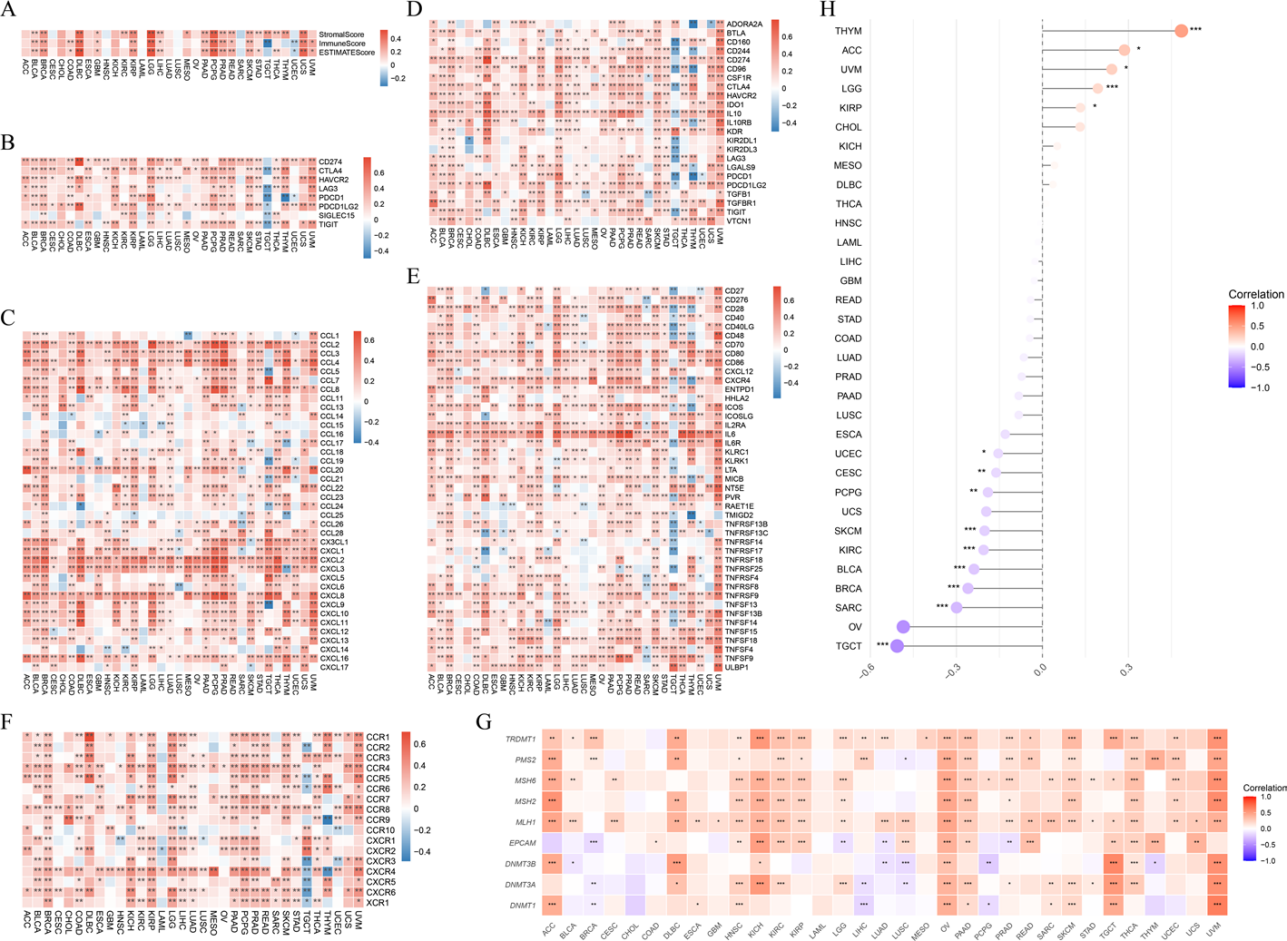
Relationship between ATF3 expression and immunity/differentially methylated probes-based stemness index (DMPsi). **A** ESTIMATE analysis of the association between ATF3 expression and ImmuneScore, StromalScore, and EstimateScore. **B**–**F** Correlations of ATF3 expression with immune-checkpoint genes (**B**), chemokines (C), immune inhibitors (D), immune stimulators (E) and chemokines receptors (F). **G** Correlations between ATF3 and mismatch-repair and DNA-methyltransferase genes. **H** Correlation between ATF3 and DMPsi. *, *P* < 0.05; **, *P* < 0.01; ***, *P* < 0.001

ESTIMATE analysis revealed that in most cancer types, ATF3 expression had a positive connection with ImmuneScore, StromalScore, and EstimateScore, indicating that tumors with high ATF3 expression have a more complex TME (Fig. 4A). The strongest correlations were observed in diffuse large B-cell lymphoma (DLBC), LGG, and pheochromocytoma and paraganglioma (PCPG). However, an opposite trend was observed in testicular germ cell tumors (TGCT), where ATF3 had a substantial negative connection with ImmuneScore. Further investigation unveiled that ATF3 was positively connected with the expression of the vast majority of ICGs, chemokines and their receptors, immune stimulators, and immune inhibitors in most tumor types, such as LGG, UVM, and BRCA, but opposite trends were also observed in TGCT (Fig. 4B-F). Moreover, ATF3 expression exhibited a strong connection with the mismatch repair genes and DNA methyltransferases in most cancer types, which was particularly pronounced in UVM (Fig. 4G). Tumor stemness analysis indicated that ATF3 expression had the strongest correlation in THYM and the strongest negative correlation in TGCT (Fig. 4H). In summary, the contribution of abnormal ATF3 expression to the TME was significant.

### ATF3 was downregulated in KIRC and associated with better prognosis

**Fig. 5:**
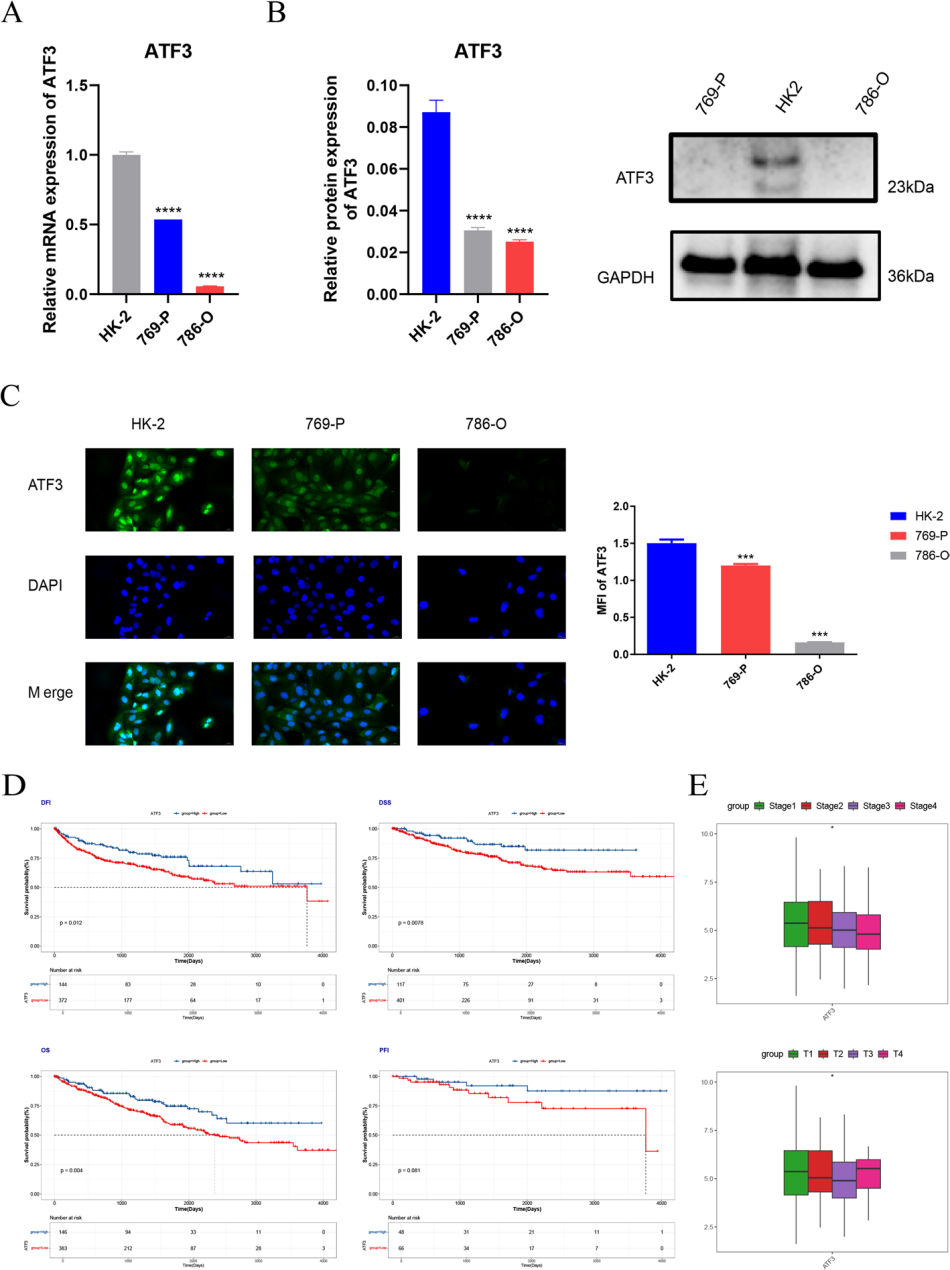
ATF3 was down-regulated in kidney clear cell renal cell carcinoma (KIRC) and associated with favorable prognosis. **A**–**C** ATF3 expression in KIRC cell lines determined by real-time quantitative polymerase chain reaction (RT-qPCR) (**A**), Western blot (**B**) and immunofluorescence (**C**). **D** Kaplan-Meier survival curves comparing high vs. low ATF3 expression for DFI, DSS, OS and PFI in KIRC. **E** Association between ATF3 and clinical-feature subgroups in KIRC. *, *P* < 0.05; ***, *P* < 0.001; ****, *P* < 0.0001

The prior examination examined the function of ATF3 in pan-cancer, followed by an investigation into the involvement of ATF3 in KIRC. RT-qPCR, Western blot, and immunofluorescence experiments unveiled that ATF3’ expression in KIRC cell lines (769-P and 786-O) were significantly downregulated, consistent with the bioinformatics analysis results (Fig. 5A-C). Clinical data analysis unveiled that elevated ATF3 expression was substantially connected with better OS, DFI, and DSS in KIRC patients (*P* < 0.05) (Fig. 5D), and its expression was markedly connected with stage and T stage (*P* < 0.05) (Fig. 5E).

**Fig. 6:**
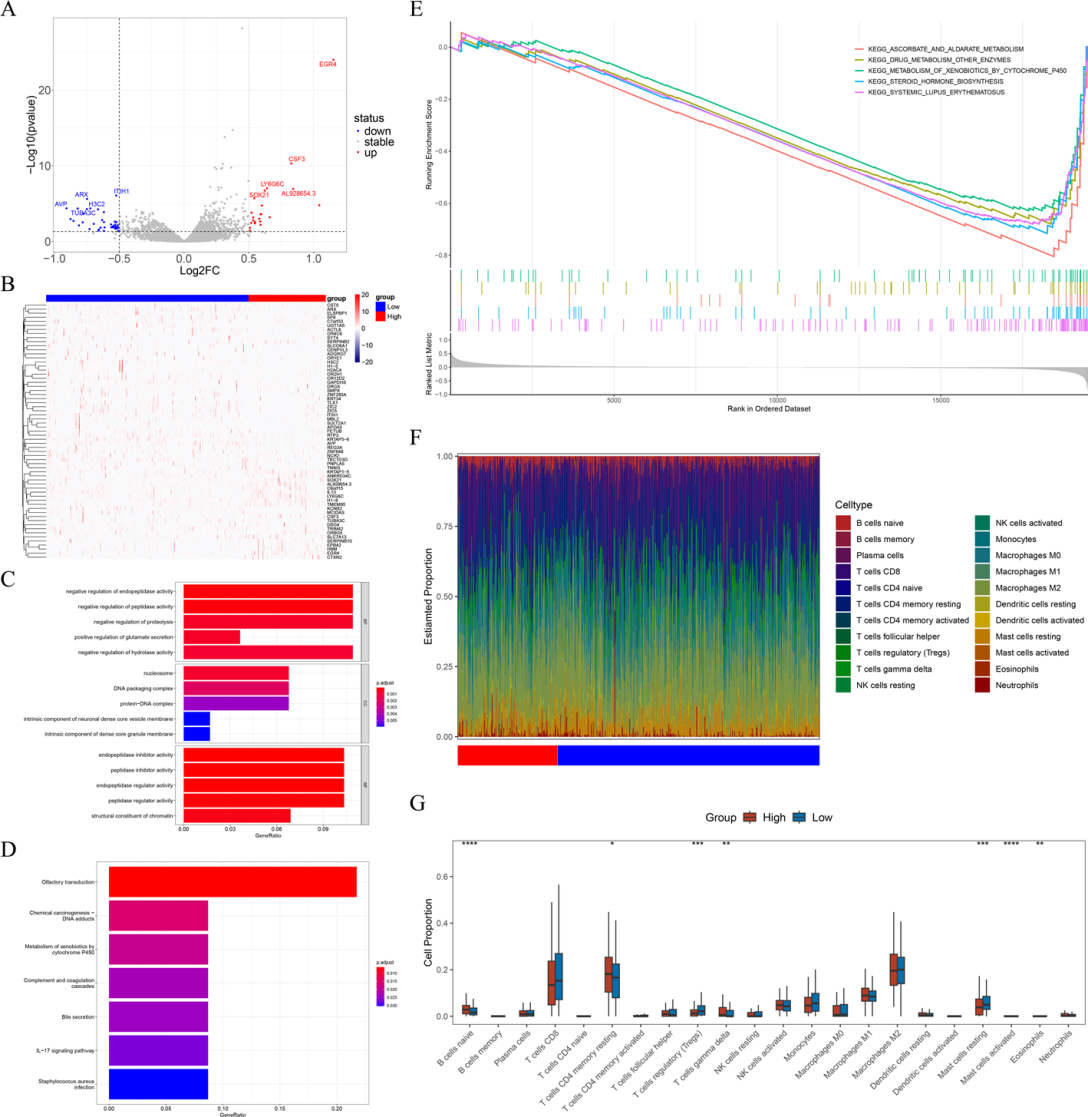
Enrichment and immune-infiltration analyses of ATF3. **A** Volcano plot of differentially expressed genes (DEGs) between high and low ATF3 groups. **B** Heat-map of the DEGs. **C** Gene Ontology (GO) enrichment of the DEGs. BP, Biological Process; CC, Cellular Component; MF, Molecular Function. **D** Kyoto Encyclopedia of Genes and Genomes (KEGG) enrichment of the DEGs. **E** Gene set enrichment analysis (GSEA) of ATF3. **F** Stacked bar chart of immune-cell scores in high and low ATF3 groups. **G** Box plot showing differences in immune-cell infiltration between the two groups. *, *P* < 0.05; **, *P* < 0.01; ***, *P* < 0.001; ****, *P* < 0.0001

In KIRC samples, 63 DEGs were recognized between high and low expression group (22 upregulated and 41 downregulated genes) (Fig. 6A-B). Enrichment analysis unveiled that these DEGs were mostly tied to 270 GO terms such as nucleosome, negative regulation of hydrolase activity, positive regulation of glutamate secretion, and 7 KEGG pathways such as IL-17 signaling pathway and complement and coagulation cascades (Fig. 6C-D).

### Functional and immune characteristics of ATF3 in KIRC

GSEA further indicated that ATF3 was markedly enriched in pathways encompassing ascorbate and aldarate metabolism, oxidative phosphorylation, and cytokine-cytokine receptor interaction (Fig. 6E, Table S2), suggesting that ATF3 might participate in the occurrence of KIRC by regulating metabolism and immune response. To scout the connection between ATF3 expression and the immune landscape of KIRC, immune infiltration analysis was performed. Seven immune cells—naive B cells, gamma delta T cells, regulatory T cells, active mast cells, resting mast cells, resting memory CD4 T cells, and eosinophils—showed significant variations in infiltration across the groups, revealing the immune relevance of ATF3 in KIRC (Fig. 6F-G).

### Drugs targeting ATF3 in KIRC

**Fig. 7:**
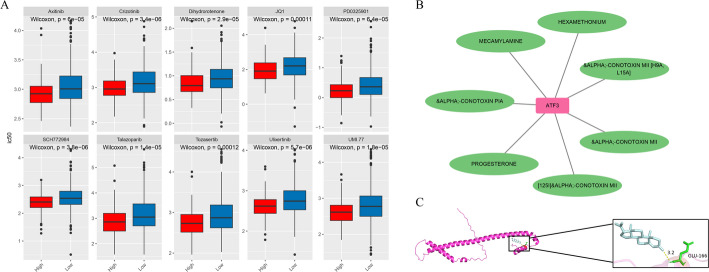
Prediction of ATF3-targeted drugs. **A** Drug-sensitivity analysis between high and low ATF3 groups (top 10 agents). **B** ATF3-drug network. **C** Molecular-docking affinity between progesterone and ATF3

Drug sensitivity analysis identified 116 significantly different anticancer drugs. Among the top 10 drugs ranked by *P*-value, axitinib, crizotinib, dihydrorotenone, JQ1, PD0325901, SCH772984, talazoparib, tozasertib, ulixertinib, and UMI.77 showed lower sensitivity in the low ATF3 expression group (Fig. 7A). We speculate that this might be closely related to the core biological functions of ATF3, such as regulating tumor immune metabolic reprogramming, oxidative phosphorylation, and tumor suppressor signaling pathways. The abnormal oxidative phosphorylation caused by the downregulation of ATF3 and the immunosuppressive microenvironment may be the key molecular basis for the decreased drug responsiveness of KIRC cells. This provides potential therapeutic suggestions for patients with KIRC. Moreover, 7 potential drugs targeting ATF3 were screened out through the DGIdb database (Fig. 7B), and two approved drugs, progesterone and mecamylamine, were selected for molecular docking. The results show that the binding energies of ATF3 to progesterone and mecamylamine are − 4.7 and − 6.9 kcal/mol (Table 1). There is one intermolecular interaction bond between the amino acid residue GLU-166 of progesterone and ATF3 (Fig. 7C), indicating that progesterone has a good binding affinity with the ATF3 protein.

**Table 1:** The molecular docking result of ATF3 with the drugs

Name	CID	Symbol	ID	Affinity(kcal/mol)	Hydrogen bonds
progesterone	4032	ATF3	AF_P18847_F1	− 6.9	1
mecamylamine	5994			− 4.7	0

### Single-cell level dissection of the cell-specific role of ATF3 in KIRC

**Fig. 8:**
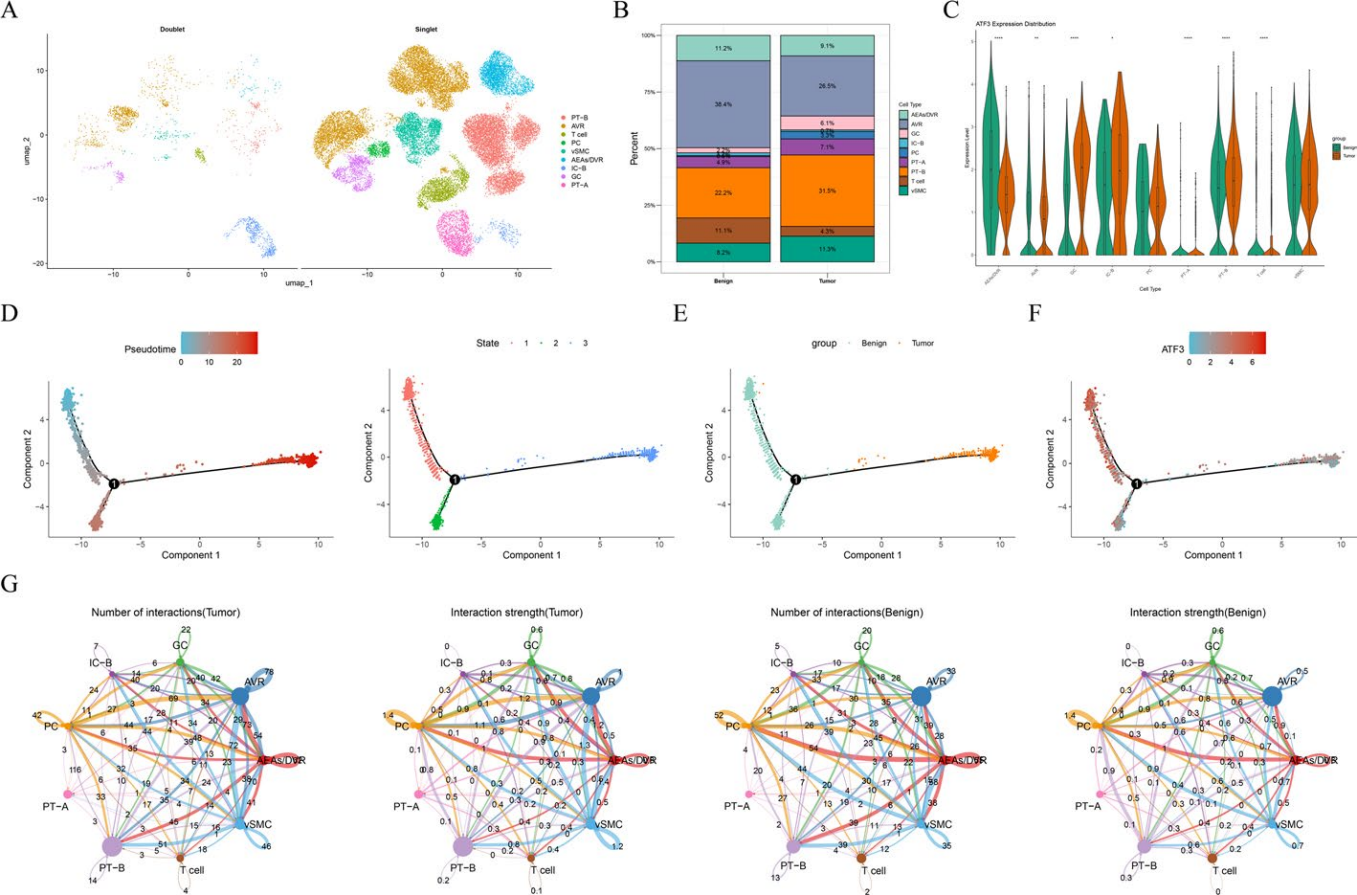
Single-cell RNA sequencing (scRNA-seq) analysis of ATF3 in KIRC. **A** Uniform Manifold Approximation and Projection (UMAP) showing cell annotations. **B** Proportions of annotated cells in KIRC vs. control. **C** ATF3 expression across cell types in KIRC vs. control. **D** Pseudotime trajectory of endothelial-afferent/efferent arterioles/descending vasa recta (AEAs/DVR) divided into three stages. **E** Distribution of KIRC and control samples along the AEAs/DVR differentiation trajectory. **F** ATF3 expression during AEAs/DVR differentiation. **G** Cell-cell communication analysis. *, *P* < 0.05; **, *P* < 0.01; ****, *P* < 0.0001

To investigate the cellular composition of KIRC at the single-cell level, quality control was performed on the scRNA-seq data (GSE159115), retaining 25,119 cells and 23,541 genes for further analysis (Figure S1A-C). The cells were divided into 14 clusters and subsequently annotated into 9 cell types (Figure S1D-E). To avoid interference with downstream analysis, 11,330 (7.5%) high-confidence doublets were removed to clarify cell boundaries (Figure S1F). These annotated cells included endothelial-afferent/efferent arterioles/descending vasa recta (AEAs/DVR), endothelial-ascending vasa recta (AVR), proximal tubule cell B (PT-B), vascular smooth muscle cell (vSMC), PT-A, endothelial-glomerular capillaries (GC), intercalated cell type B (IC-B), and principal cell (PC) (Fig. 8A). Among them, PT-B had the highest proportion in KIRC, while AVR had the highest proportion in the control group (Fig. 8B).

Based on scRNA-seq analysis, the characteristics of ATF3 in KIRC samples were detected. In AEAs/DVR, ATF3 expression was significantly downregulated in KIRC (Fig. 8C) and consistent with the expression trend of ATF3 in KIRC cells, which was defined as the key cells. Subsequently, the pseudotime trajectory of AEAs/DVR was explored, dividing the cells into three states (Fig. 8D). KIRC was mainly distributed in the late stage of AEAs/DVR, while the control was in the early differentiation stage (Fig. 8E). ATF3 was mainly expressed at the early stage of AEAs/DVR differentiation (Fig. 8F). The results of cell communication showed that communication between AVR and other cells was enhanced in KIRC, while the communication of AEAs/DVR decreased, further supporting the important role of AEAs/DVR in regulating cell-cell communication in the KIRC TME (Fig. 8G). In addition, a total of 32 driver genes that exhibited dynamic changes along the pseudo-time trajectory and were co-expressed with ATF3 were identified. Figure S2 shows the expression patterns of these dynamic genes. Analysis of the expression changes of the driver genes co-expressed with ATF3 over pseudo-time revealed that the positively correlated genes (the top 10) showed specific low expression in the early stage of the tumor, while the negatively correlated genes (the bottom 10) had a consistent expression trend throughout the two groups of samples (Figure S3).

## Discussion

KIRC is highly invasive, prone to metastasis, and associated with poor prognosis, making the identification of effective biomarkers a critical clinical priority. In this study, we found that ATF3 expression was downregulated in KIRC, and patients with high ATF3 expression had better prognosis (including OS and DFS), consistent with previous studies [47]. Therefore, ATF3 represents a promising prognostic indicator and potential tumor suppressor in KIRC.

Functional enrichment analysis revealed that ATF3 was significantly associated with immune-related pathways (including cytokine-cytokine receptor interaction and NOD-like receptor signaling pathway) and metabolism-related pathways (including oxidative phosphorylation). KIRC itself is one of the tumor types with the highest level of immune infiltration [48–50]; the characteristics of its TME not only profoundly influence disease biological behavior but also play a critical regulatory role in responsiveness to systemic therapy [51–56]. Accumulating evidence has confirmed that activation of specific metabolic pathways can reshape the TME by regulating angiogenesis and inflammatory features [57, 58]. Herein, our data suggest that ATF3 participates in this process by modulating immune cell infiltration and inflammatory responses, providing important theoretical basis for elucidating the core regulatory role of ATF3 in the KIRC immuno-metabolic network.

The cross-regulation of immunity and metabolism is the core driving force of TME remodeling, and energy metabolism reprogramming is a key switch for the functional state transition between tumor cells and immune cells [59, 60]. As a typical metabolic tumor, KIRC is characterized by core pathological features including glycolytic disorders, impaired mitochondrial oxidative phosphorylation, and abnormal lipid metabolism [14–23]. Moreover, metabolic reprogramming further mediates critical biological behaviors such as chemoresistance and maintenance of cancer stem cell properties [24–26], indicating that the discovery of metabolic regulatory targets is an important direction for KIRC precision therapy. Oxidative phosphorylation, as a mitochondrial-dependent and highly efficient energy metabolism pathway, is closely related to the survival, activation, and invasion capabilities of immune cells [61, 62]. This study suggests that the regulation of the oxidative phosphorylation pathway by ATF3 is not limited to maintaining energy homeostasis, but rather directly affects the metabolic phenotype and functional differentiation of T cells by modulating the activity of key enzymes in the mitochondrial respiratory chain and mitochondrial biogenesis. Under physiological conditions, high ATF3 expression sustains the oxidative phosphorylation metabolic preference of effector T cells, ensuring sufficient ATP production to support proliferation, cytokine secretion, and cytotoxic functions [63, 64]. In contrast, when ATF3 is downregulated in KIRC, oxidative phosphorylation in effector T cells within the TME is suppressed, forcing a metabolic switch toward glycolysis. However, due to the far lower glycolytic efficiency compared to tumor cells, effector T cells undergo functional exhaustion due to energy depletion, characterized by reduced proliferation, impaired cytotoxicity, and ineffective tumor tissue infiltration [20, 22, 65]. Meanwhile, abnormal oxidative phosphorylation induced by ATF3 downregulation also affects T cell epigenetic modifications, inhibiting the expression of effector-related transcription factors and further exacerbating effector T cell functional inactivation [14, 16, 66]. Our immune infiltration analysis demonstrated significant differences in the infiltration levels of various cell types between the high and low ATF3 expression groups, indicating that ATF3 may provide necessary metabolic support for the infiltration and function of anti-tumor immune cells by maintaining energy metabolic homeostasis, thereby enhancing the body’s immune surveillance against tumors. This result is consistent with the core regulatory role of ATF3 in tumor metabolism, suggesting that ATF3 may be a key molecule regulating KIRC metabolic reprogramming. Its downregulation may directly induce core metabolic abnormalities such as impaired mitochondrial oxidative phosphorylation in KIRC, while further exacerbating the immunosuppressive state of the TME through immuno-metabolic cross-regulation.

The present study confirmed that ATF3 is closely associated with the IL-17 signaling pathway, which serves as a core hub for ATF3-mediated immune regulation in KIRC, mediating the abnormal infiltration of regulatory T cells (Treg) through a “dual immuno-metabolic regulation” mechanism. In terms of immune regulation, ATF3 can directly bind to the promoter regions of key genes in the IL-17 pathway and inhibit their transcriptional activation. When ATF3 is downregulated in KIRC, this inhibitory effect is abrogated, leading to excessive activation of the IL-17 signaling pathway and triggering chronic inflammatory responses in the TME. Additionally, IL-17 can directly promote the proliferation of intrinsic Treg in the TME, further increasing the Treg infiltration ratio [67, 68]. In terms of metabolic regulation, IL-17 pathway activation mediated by ATF3 downregulation forms a synergistic regulatory network with abnormal oxidative phosphorylation: IL-17 can further block effector T cell oxidative phosphorylation by inhibiting the expression of mitochondrial pyruvate carriers, exacerbating their energy depletion and functional exhaustion. Meanwhile, IL-17 promotes glycolytic metabolic reprogramming in tumor cells and Treg, endowing Treg with superior metabolic competitiveness compared to effector T cells, enabling their survival and immunosuppressive functions in the nutrient-depleted TME [69, 70]. These results indicate that the ATF3-IL-17 axis exerts a multi-dimensional regulatory mechanism on Treg infiltration. Furthermore, the expression level of ATF3 was significant correlated with complement and coagulation cascades, further suggesting that it may participate in the remodeling of the TME by regulating the cross-reactions between nonspecific immunity and metabolism. Activation of the complement system can not only directly kill tumor cells, but also affect immune cell infiltration by regulating cytokine release [71, 72]. The metabolic requirements during its activation process may be associated with the ATF3-regulated oxidative phosphorylation pathway [73, 74]. Collectively, these findings suggest that ATF3 serves as a potential core regulatory factor in the multi-dimensional regulatory network connecting immune function, metabolic reprogramming, and tumor progression in KIRC.

Proximal arterioles (AEAs) and distal vascular reducts (DVRs) are key structures for sustaining glomerular filtration rate and renal interstitial oxygenation. The AEA, positioned at the glomerular inlet, modulates blood flow into the glomerulus, whereas the DVR, placed surrounding the renal tubules, contributes to the maintenance of oxygen delivery to the renal interstitium and supports the metabolic requirements of the kidney [75, 76]. The endothelial cells of the AEA/DVR form the inner lining of blood arteries and regulate local hemodynamics and vascular tone by secreting numerous vasoactive chemicals, including nitric oxide, prostaglandins, and endothelin [77]. Additionally, these endothelial cells secrete inflammatory mediators, contributing to local inflammatory responses and immunological control, so fulfilling a dual function in preserving renal homeostasis [77]. Notably, the single-cell analysis demonstrated that ATF3 expression in AEA/DVR endothelial cells is significantly downregulated in KIRC. Given that ATF3 is a key factor expressed during the early differentiation stages of AEA/DVR, its downregulation suggests potential disruption of vascular development/homeostasis programs in KIRC. Considering the central role of AEA/DVR in hemodynamics and immune regulation, we hypothesize that the loss of ATF3 expression may disrupt the normal function and immune regulatory capacity of tumor-associated vasculature. However, whether ATF3 regulates immune cell infiltration and inflammatory responses by influencing the activation of the AEA/DVR endothelial cell metabolic pathway, and thereby regulating tumor angiogenesis and the local inflammatory microenvironment, remains to be further studied in the future. Additionally, combined with the regulatory role of the TME in KIRC treatment response, the ATF3-mediated immuno-metabolic-angiogenesis regulatory network may also be an important factor affecting the efficacy of systemic therapy in KIRC, and its clinical translational value warrants further validation.

Drug sensitivity analysis further revealed the clinical translational value of ATF3 biological functions. Axitinib, a classic multi-target tyrosine kinase inhibitor, exerts anti-tumor effects mainly by inhibiting the VEGFR/PDGFR/EGFR signaling pathway to block tumor angiogenesis and cell proliferation [78]. Notably, previous studies have confirmed that ATF3 can directly inhibit the EGFR/AKT/GSK3β/β-catenin signaling pathway in KIRC, and downregulation of ATF3 expression leads to abnormal activation of the EGFR/AKT pathway. This activation not only promotes the proliferation and invasion of KIRC cells but also compensates for the inhibitory effect of axitinib on the EGFR pathway, resulting in KIRC cell desensitization to axitinib [29]. Furthermore, ATF3 downregulation inhibits oxidative phosphorylation in the TME and triggers immune metabolic reprogramming, further exacerbating hypoxia and nutrient depletion in the KIRC TME. Meanwhile, ATF3 downregulation mediates excessive activation of the IL-17 signaling pathway and a significant increase in Treg infiltration, forming a highly immunosuppressive TME [14, 16]. This TME not only impairs the anti-tumor immune response but also reduces drug accumulation in tumor tissues by regulating tumor vascular permeability and inhibits drug-induced tumor cell apoptosis by secreting immunosuppressive factors, thereby further decreasing the sensitivity of the low ATF3 expression group to drugs such as axitinib.

This study adopted an integrated strategy combining pan-cancer analysis, single-cell sequencing, and bioinformatics enrichment analysis. This approach is highly consistent with the current research trends in the field of oncology, such as multi-omics integration and exploration of cross-disease mechanisms [7, 8], and provides a more systematic perspective for the screening of biomarkers in KIRC. This study found that ATF3 expression was significantly downregulated in KIRC, and it may play a key role in KIRC progression by regulating the immune-metabolic network and tumor vascular function, providing new theoretical support for establishing ATF3 as a potential biomarker or therapeutic target for KIRC. Although these findings offer significant insights, the limitations of our investigation must be acknowledged. First, although our conclusions were partially verified by RT-qPCR, Western blot, and immunofluorescence, large-scale clinical validation and in-depth mechanistic experiments were not performed in the present study. Second, the study failed to fully account for the impact of cellular heterogeneity, and its conclusions require validation in larger clinical cohorts. Third, future research should clarify the specific mechanisms by which ATF3 regulates endothelial cell activity and the tumor microenvironment through in vivo and in vitro functional experiments. It should also systematically evaluate its clinical translational potential as a prognostic indicator or treatment response predictor, thereby providing new insights for precision diagnosis and treatment of KIRC.

## Conclusion

In conclusion, this study confirms that ATF3 is downregulated, exhibits prognostic significance, and elucidates its role in regulating the immune-metabolic network and tumor angiogenesis in KIRC. These findings support ATF3 as a potential therapeutic target for KIRC, providing new insights into precision diagnosis and treatment strategies for this malignancy. Future research requires larger-scale clinical cohort studies and functional experiments to validate the clinical translational potential of ATF3 and elucidate its specific regulatory mechanisms in KIRC progression.

## Supplementary Information

Below is the link to the electronic supplementary material.

Supplementary Material 1.

Supplementary Material 2.

Supplementary Material 3.

Supplementary Material 4.

Supplementary Material 5.

## Data Availability

The data for this study were sourced from UCSC Xena (https://xenabrowser.net/datapages/) and Gene Expression Omnibus (GEO) (GSE159115, http://www.ncbi.nlm.nih.gov/geo/).

## Abbreviations

KIRC: kidney renal clear cell carcinoma
RT-qPCR: real-time quantitative polymerase chain reaction
TCGA: The Cancer Genome Atlas
GEO: Gene Expression Omnibus
ROC: receiver operating characteristic
KM: Kaplan-Meier
OS: overall survival
PFI: progression-free interval
DFI: disease-free interval
DSS: disease-specific survival
MSI: microsatellite instability
TMB: tumor mutation burden
DMPsi: differentially methylated probes-based stemness index
ICGs: immune checkpoint genes
DEGs: differentially expressed genes
FC: fold change
GO: Gene Ontology
KEGG: Kyoto Encyclopedia of Genes and Genomes
GSEA: Gene Set Enrichment Analysis
IC_50_: inhibitory concentration
UMAP: Uniform Manifold Approximation and Projection
SD: standard deviation
TME: tumor microenvironment
