# Supplementary material for: Comprehensive analysis of ATF3 as a diagnostic and prognostic biomarker from pan-cancer to clear cell renal cell carcinoma
Source: Discov Oncol. 2026 Apr 30;17:657. doi: 10.1007/s12672-026-05113-x (PMC13129119; doi:10.1007/s12672-026-05113-x)
Supplement: Supplementary file 1 — Supplementary Material 1. [file 12672_2026_5113_MOESM1_ESM.docx]

**
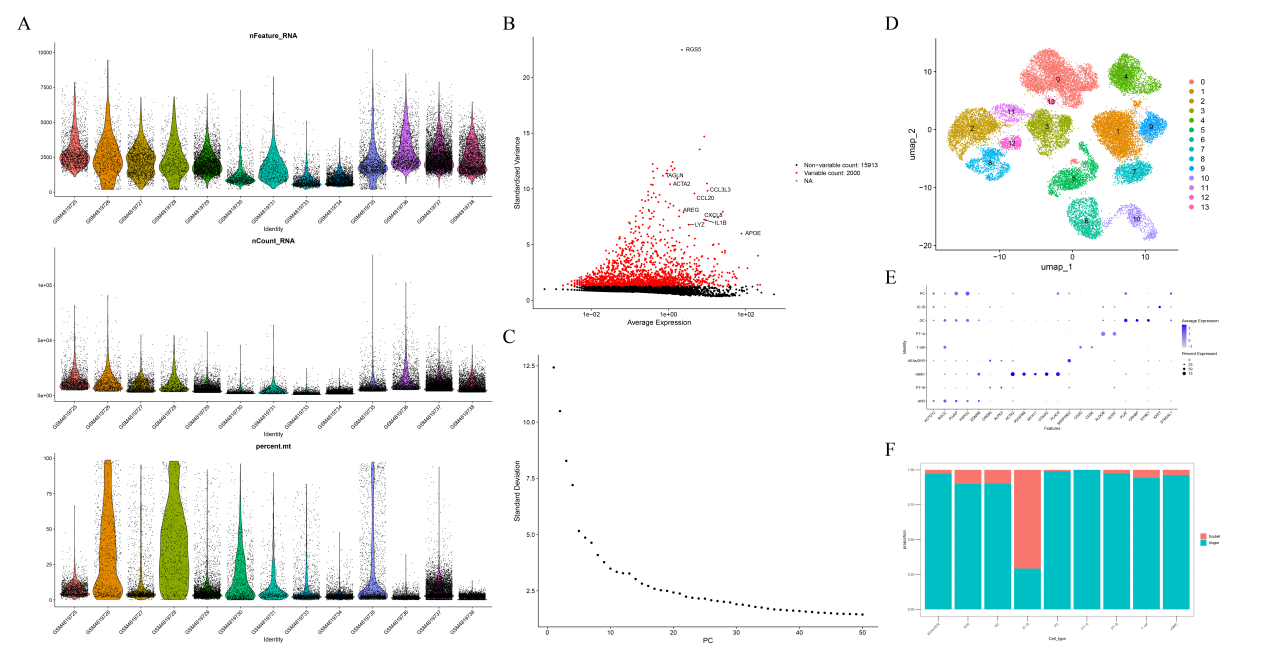
**

**Figure S1 Filtering of single-cell RNA-seq (scRNA-seq) data. (A)** Quality control metrics. **(B)** Top 2000 highly variable genes were selected. **(C)** Principal component analysis; the first 30 principal components were used. **(D)** Uniform Manifold Approximation and Projection (UMAP) visualization of single-cell clusters. **(E)** Cell-type annotation with marker genes. **(F)** Percentage of cell doublets.
