# Supplementary material for: Comprehensive analysis of ATF3 as a diagnostic and prognostic biomarker from pan-cancer to clear cell renal cell carcinoma
Source: Discov Oncol. 2026 Apr 30;17:657. doi: 10.1007/s12672-026-05113-x (PMC13129119; doi:10.1007/s12672-026-05113-x)
Supplement: Supplementary file 2 — Supplementary Material 2. [file 12672_2026_5113_MOESM2_ESM.docx]

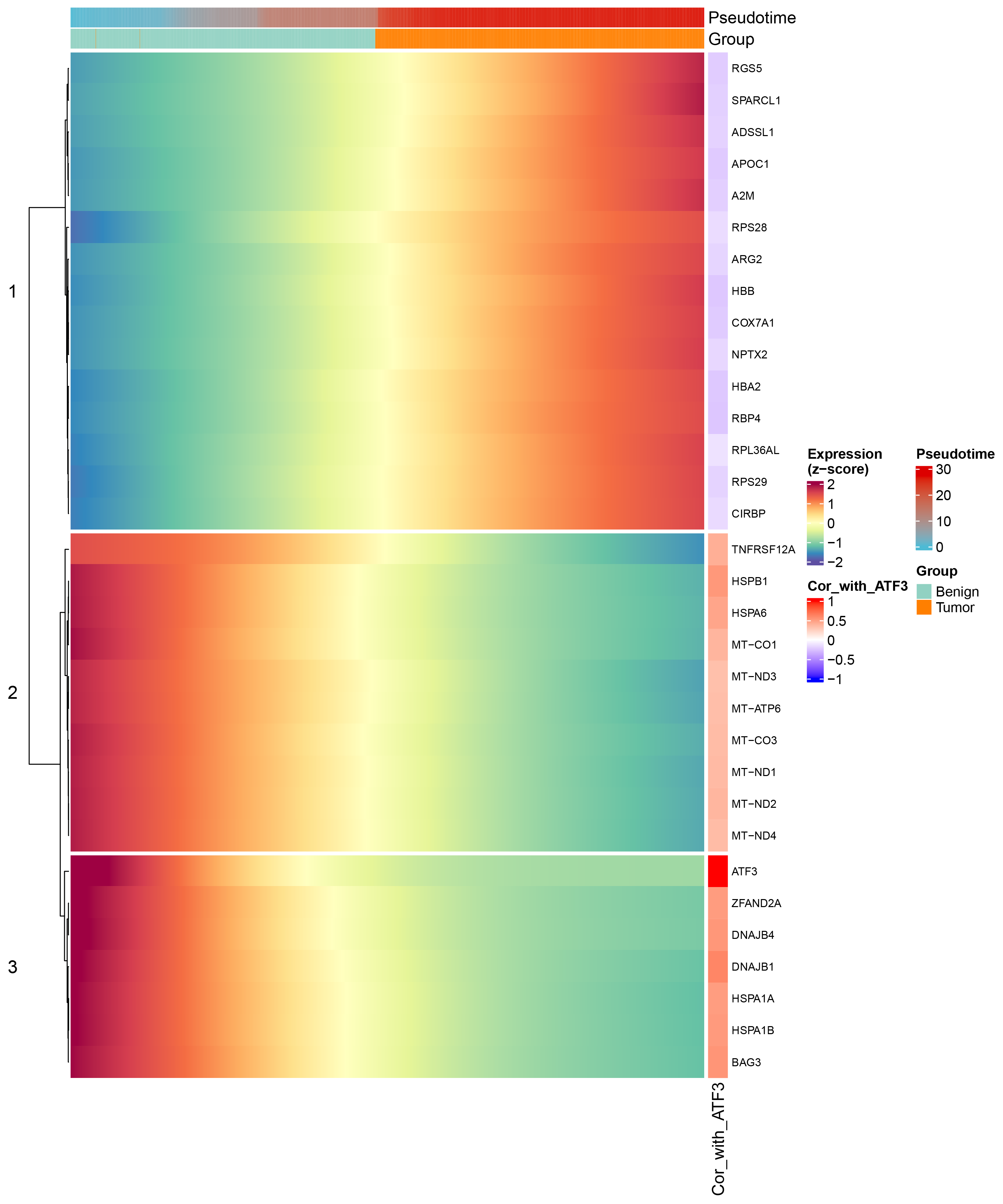


**Figure S2 Expression pattern of the driver genes co-expressed with ATF3 in the heatmap.** Rows represent genes, clustered according to expression patterns; columns represent cells, sorted by pseudo-time. Top annotation bars display pseudo-time values and sample groups. Right annotation bars show the Spearman correlation coefficient of each gene with ATF3.


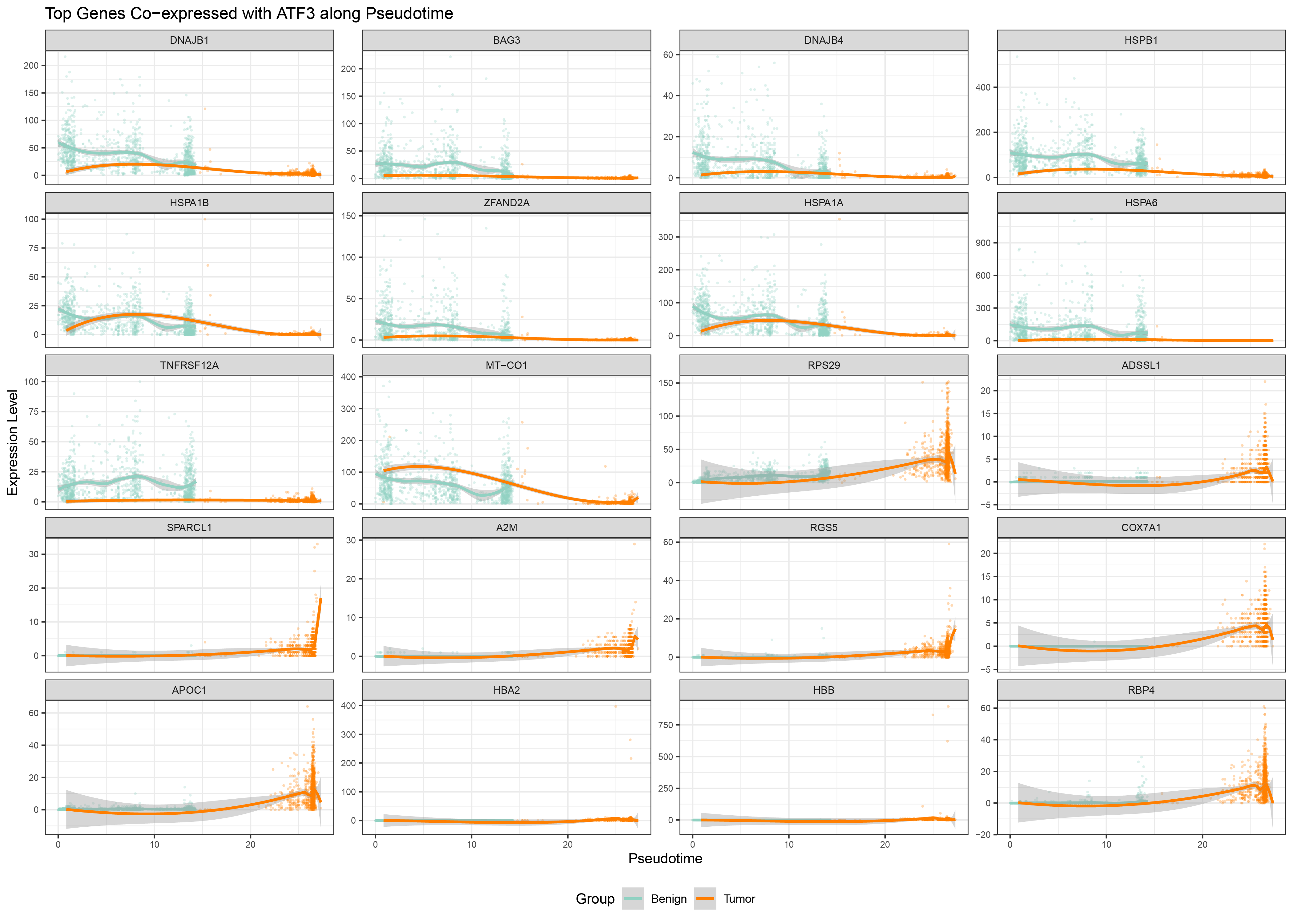


**Figure S3 The expression levels of the driver genes that co-express with ATF3 change over pseudo-time.** The scattered dots represent individual cells. The green ones represent the benign group, and the orange ones represent the tumor group.
