# Supplementary material for: Comprehensive analysis of ATF3 as a diagnostic and prognostic biomarker from pan-cancer to clear cell renal cell carcinoma
Source: Discov Oncol. 2026 Apr 30;17:657. doi: 10.1007/s12672-026-05113-x (PMC13129119; doi:10.1007/s12672-026-05113-x)
Supplement: Supplementary file 3 — Supplementary Material 3. [file 12672_2026_5113_MOESM3_ESM.docx]

Raw western-blot image


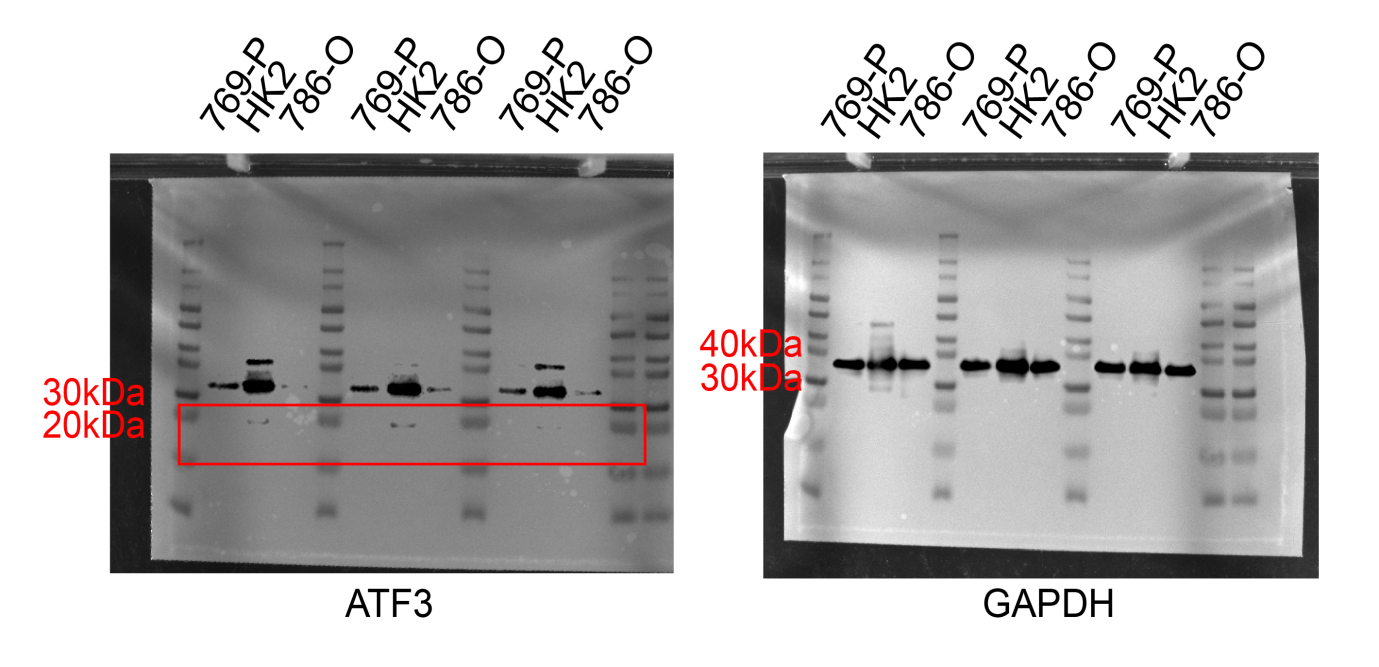


**Figure. Original images for Western blotting for ATF3 (23kDa) and GAPDH (36kDa) in figure 5.**
