# Supplementary material for: Comprehensive analysis of ATF3 as a diagnostic and prognostic biomarker from pan-cancer to clear cell renal cell carcinoma
Source: Discov Oncol. 2026 Apr 30;17:657. doi: 10.1007/s12672-026-05113-x (PMC13129119; doi:10.1007/s12672-026-05113-x)
Supplement: Supplementary file 4 — Supplementary Material 4. [file 12672_2026_5113_MOESM4_ESM.docx]

Table S1 The list of primers used for this study

| Primer name | Sequence 5'-3' |
| --- | --- |
| GAPDH | F:5'-CTGGGCTACACTGAGCACC-3' |
|  | R:5'-AAGTGGTCGTTGAGGGCAATG-3' |
| ATF3 | F:5'-CCTCTGCGCTGGAATCAGTC-3' |
|  | R:5'-TTCTTTCTCGTCGCCTCTTTTT-3' |
